# Phytoaccumulation of heavy metals from municipal solid waste leachate using different grasses under hydroponic condition

**DOI:** 10.1038/s41598-020-72800-2

**Published:** 2020-09-25

**Authors:** Malik Muhammad Hassan, Noor Haleem, Muhammad Anwar Baig, Yousuf Jamal

**Affiliations:** Institute of Environmental Sciences and Engineering (IESE), School of Civil and Environmental Engineering (SCEE), National University of Sciences and Technology (NUST), Islamabad, 44000 Pakistan

**Keywords:** Biotechnology, Plant sciences, Environmental sciences

## Abstract

Grasses have been used widely to remediate contaminants present in domestic wastewater, but leachate generated from municipal solid waste that usually contain some concentrations of heavy metals has never been reported to be treated with grasses, especially Rhodes grass. A series of experiments was performed to investigate the contaminant uptake from municipal solid waste leachate by *Chloris gayana* (Rhodes grass) grown in combination with two commonly available grass varieties namely *Vetiveria zizanioides* (Vetiver grass) and *Pennisetum purpureum* (Elephant grass). Leachate used for the experiments had high values for chemical oxygen demand (5 g/L), pH (8.5), electrical conductivity (9.0 mS/cm), nitrates (182.1 mg/L), phosphates 6.4 mg/L along with heavy metals i.e. copper, zinc and manganese. Different dilutions of leachate ranging from 0 to 100% were applied in batches and their result showed that collectively all the grasses reduced overall contaminant concentrations. These were reported for chemical oxygen demand, electrical conductivity, nitrates, and phosphates reduced up to 67, 94, 94, and 73%, respectively. Metals uptake by grasses also showed a significant decrease in applied dose i.e. zinc (97%), copper (89%), and manganese (89%). Plant analysis showed that all grasses showed preference to heavy metals uptake e.g. Rhodes grass favoured up taking zinc, Elephant grass for copper and Vetiver grass preferred manganese. Overall growth performance of Rhodes grass was better in dilute leachate, whereas in more concentrated leachate, Rhodes grass did not perform better and production of biomass decreased. In Vetiver grass, root and shoot lengths decreased with increasing leachate strength, but the biomass did not change significantly.

## Introduction

Open dumping of municipal solid waste is a major problem in developing countries^1^ owing to environmental pollution (air, land and water), health hazards, vegetation loss and unpleasant odour. These hazards are mainly a consequence of biodegradation due to putrescible nature of solid waste at the dumpsites, which generates leachate^2,3^. If the leachate goes unattended by percolation into subsoil layers, it can deteriorate the underlying soil and groundwater^4^. The quantity and quality of leachate depends upon the source of water entering into the dumpsite^5^.

Leachate is known for having high concentrations of organics, nutrients and heavy metals^6^. Among these contaminants, metals are rated as the most threatening contaminant that can deteriorate the groundwater quality to the level of being hazardous^7^. The main sources of heavy metals in leachate are batteries, electronic waste, pesticides, photographic chemicals, personal care products, certain detergents, fluorescent tubes, waste oil, pharmaceuticals, wood treated with dangerous substances and the paints^8^.

The organic component leaches down from the partially decomposed organic material present in waste. The decomposition leads to mineralization of organic compounds and affect the solubility of various compounds. In the early stages of decomposition, organics are usually complex (e.g. lignin and cellulose) but with decomposition these transform into humic and fulvic acid like substances, which are comparatively more mobile than the complex compounds^9^. Metals adhere to the fragments of these organics and help them to dissolve in water. This process leads to high metals concentrations in leachate. If there is not leachate retention or collection system, the metals accumulate in topsoil and stay there for extended time period. Metals are also mixed with inert portion of solid waste and daily soil cover being added at the dumping site.

Different methods are currently being used for treatment of municipal solid waste leachate which include biological, physical and chemical treatments. These methods are further categorised as aerobic biological (treatment such as aerated lagoons and activated sludge), anaerobic biological treatment (such as anaerobic lagoons and reactors), physiochemical treatment (such as air stripping, pH adjustment, chemical precipitation, oxidation and reduction), Coagulation (using lime, alum, ferric chloride) and advanced techniques (such as carbon adsorption and ion exchange)^10,11^. Among these techniques phytoremediation is a natural method for stabilizing and desalinizing leachate. Phytoremediation is a technique that offers multiple contaminants removal at a natural manner with minimal costs^12^.

Vetiver grass (*Vetiveria zizanioides* lately named as *Chrysopogon zizanioides*), is reported in literature to be widely used for metals removal from leachate^7^. It is tall (1–2.5 m), fast growing, tolerant to heavy metals and with its lengthy root system, it creates massive root system beneath the soil^13^. Elephant grass (*Penisetum purpurium Schumach*) is also reported for phytoremediation purposes, owing to its excessive production of cellulose biomass, tolerance to heavy metals especially copper and high growth rates^14–17^. However, Rhodes grass (*Chloris guyana*) has never been tried for remediating leachates. This grass has high remediation capacity, it is also tolerant to adverse climates and shows high salt tolerance^18^.

In order to study the performance of various grasses experiments were conducted at the Institute of Environmental Sciences and Engineering (IESE), National University of Sciences and Technology (NUST), Islamabad, Pakistan under hydroponic environment to investigate the treatment of leachate contaminants and metal uptake by the above mentioned three grass species in combination. The objectives of the study were to evaluate the reduction of contaminants, nutrients uptake and metals removal from municipal solid waste leachate using three grasses and compare their growth performance at various leachate dilution levels. It was hypothesised that the efficiency of metal removal from leachate is increased by Vetiver, Elephant, and Rhodes grass grown in combination and metal uptake will decrease the growth of the grasses due to toxic effect.

## Materials and methods

### Collection of grasses

Three types of grasses (Vetiver, Elephant, and Rhodes) were taken from the nursery of National Agriculture Research centre (NARC), Islamabad, Pakistan. The plants were two-month old with shoot and root of 15 and 9 cm length for *Chrysopogon zizanioides* (Vetiver grass), 12 and 5 cm for *Pennisetum purpureum* (Elephant grass) and in case of *Chloris gayana* (Rhodes grass) the shoot and root were 10 and 6 cm long respectively.

### Leachate characteristics

Fresh leachate was collected in 50 L jerry cans from Losar Dumpsite (official unscientific open dumpsite of Rawalpindi city, Pakistan) and brought to IESE laboratories for their chemical and heavy metal analysis and later on use for growing grasses in the hydroponic tubs at various dilutions. This dump site receives daily 1,200 metric tons of municipal solid waste which contains major proportion > 50% as organic waste^19^.

#### Leachate analysis

Leachate analysis were performed to note basic parameters like electrical conductivity (EC) and pH using conductivity and pH meters (ino Lab pH/Cond 720). Nitrates and phosphates contents in the leachate were determined before and after the experiments of grass growing using UV–Visible Spectrophotometer (T-60 PG Instruments: Wavelength Range: 190–1100 nm)^20^. Atomic Absorption Spectroscopy (AAnalyst 800, Perkin Elmer) was used to measure heavy metals concentration present in leachate. Chemical Oxygen Demand (COD), Nitrate & Phosphate were determined using standard open flux method 5220 B, 4500 NO_3_^−^ B ASTM & 4500-P C ASTM. Leachate was diluted to bring variation in leachate strength and analyse the grasses tolerance in leachate strength. 10 dilutions in different pots were made. Total volume was determined 6 litter and in 10% 5.4 L distilled water and 0.6 L leachate was added. Same calculation was carried out in all other (20, 30, 40, and 100%) dilution. The results of these dilutions for different parameters are given in Table 1. While, Table 2 shows the mass balance of metals at the inflow and outflow conditions.

**Table 1:** Analysis of leachate parameters at different dilutions.

Parameters	Units	Treatments									
		10	20	30	40	50	60	70	80	90	100
COD	g/L	3.46	4.16	4.45	4.68	4.93	5.28	5.64	5.96	6.14	6.93
pH	–	8.01	8.16	8.27	8.41	8.58	8.67	8.77	8.84	8.89	8.92
EC	mS/cm	3.08	5.04	7.41	9.07	10.16	10.64	11.07	13.16	13.91	14.28
NO_3_^-^	mg/L	92.9	104.6	108.0	146.4	172.2	176.0	207.0	249.0	267.0	295.0
PO_4_^3-^	mg/L	2.40	3.30	4.20	4.50	5.10	6.10	7.10	9.20	10.30	11.30
Mn	mg/L	44.95	57.52	75.00	90.00	108.0	124.0	137.0	154.0	170.0	190.0
Cu	mg/L	51.86	60.28	70.9	79.71	97.12	101.0	111.0	120.0	133.0	156.0
Zn	mg/L	73.85	94.03	107.0	123.0	129.0	183.0	207.0	239.0	312.0	378.0

**Table 2:** Mass balance for three heavy metals used in experiment.

Metals		Grass	Mn	Cu	Zn	Total (%)
Inflow (mg)			115.21	98.10	184.95	
Uptake (mg)		Rhodes	34.20	26.50	75.77	45
		Vetiver	44.10	32.50	60.23	46
		Elephant	34.40	37.60	45.27	39
Outflow (mg)			2.51	1.50	3.68	
Percentage uptake		Rhodes	29.68	27.01	40.97	33
		Vetiver	38.28	33.13	32.57	35
		Elephant	29.86	38.33	24.48	31

#### Leachate strength (%)

Leachate of various strength was prepared using distilled water dilution. In all eleven leachate strength solutions were prepared and maintained as 10% to 100% with a step of 10%. Total volume of diluted solution prepared for these experiment was 6 L.

### Hydroponic experiment

**Figure 1:**
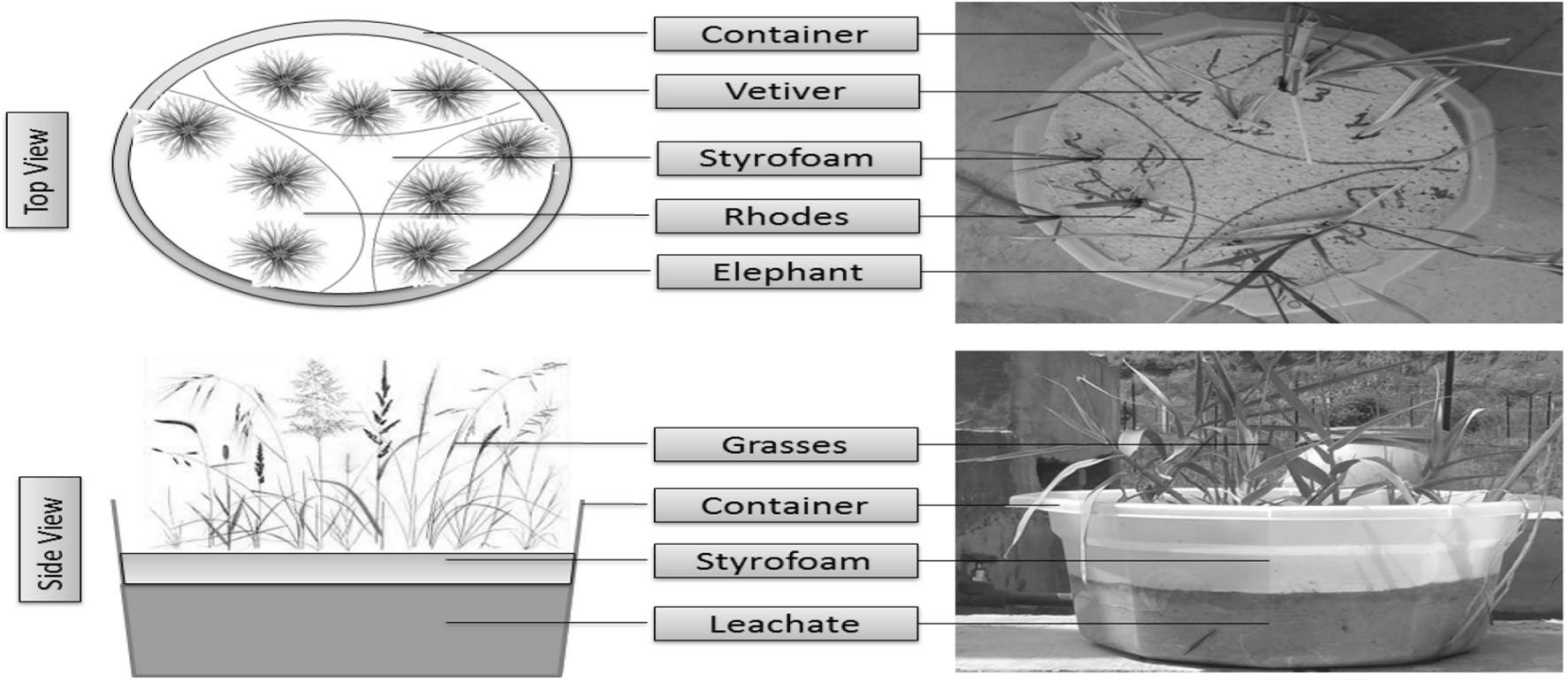
Experimental setup.

For this purpose, eleven HDPE/PVC pots as shown in Fig. 1 measuring 27.94 cm diameter and 15.24 cm height were used for these experiments. Eleven dilutions of leachate as given in table-1 were prepared (11th is distilled water) and 6 litter volume was filled into each pot. Equal number of plants (3 plants of each grass type with 9 in total) were placed in Styrofoam sheet as floating bed grown in each pot as shown in Fig. 1. The area was divided into three zones with three plants in a group of individual grass to be fed with single concentration of leachate in order to observe the efficiency of grass species as shown.

### Plant analysis

After growing the shoots for four months, the plant samples were washed with tap water followed by deionized water and their shoots and roots were cut and placed separately. Plants height was measured at the time of plantation and harvesting whereas length of roots and shoots were measured separately. Moreover, weight of shoots and roots was measured before and after drying grasses in oven at 60 °C for 72 h. Then the dried biomass was weighed and fine ground in pestle and mortar^21^. For Cu, Zn and Mn extraction, 100 mg of plant material was digested with a mixture of 97.9% HClO_4_ & 69% HNO_3_ (2:1 v/v) in a microwave digester then solution heated until the brown fumes disappear^22^. It was then cooled and 5 mL of diluted (1:1) HCl (density 1.18 g/mL) added, and finally diluted with H_2_O up to 25 mL solution. Then the sample was filtered using the filter paper of 1 µm and the filtrate was diluted for Atomic Absorption Spectroscopy^23^. Concentration of Cu, Zn and Mn were found through Flame Atomic Absorption Spectrometer. Moreover, the shoot/root metal concentration ratio, was also calculated.

## Results and discussion

### Plants height

**Figure 2:**
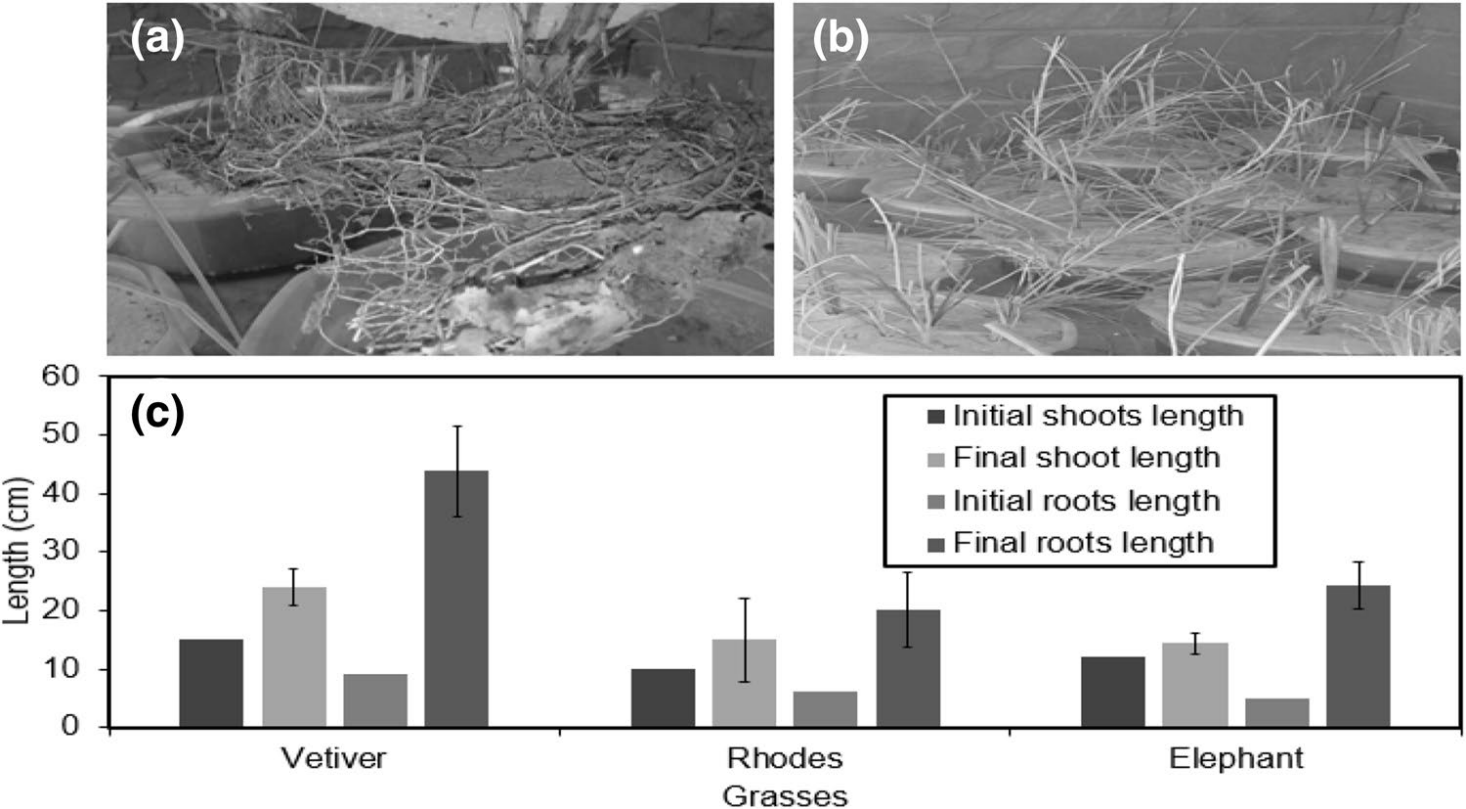
The initial and final roots and shoots length of (**a**) Vetiver (**b**) Rhodes and (**c**) Elephant grass. (For the period of four months in hydroponic environment).

At the time of planting in the start of experiment, grasses were grown with following root and shoot length i.e. initial length s of shoots were 15, 10, 12 (cm) and roots measuring 9, 6, and 5 (cm) for the Vetiver, Rhodes and Elephant grass respectively. After keeping growing in the respective leachate dilutions for a period of four months, various grass species responded to treatment differently. The length of roots and shoots was found to increase many folds as shown in Fig. 2. The plants were harvested after 4 months and the root, shoot length along with weight was noted as shown in Fig. 2.

Figure 2a is showing roots pattern of all grasses grown in tubs they were noted long in length but round in shape due to growth in tub. Figure 2b expresses the shoots trend of all grasses. Shoots growth was noted 2–3 time higher than their initial heights. Figure 2c shows the mean and standard deviation of final shoots and roots length. The final results of shoots and roots of Vetiver grass showed good and final length grown to 44 and 24 cm respectively. In case of Rhodes grass roots and shoots results as compared to the other grasses. Vetiver grass roots and shoots initial length was 9 and 15 cm length at initial level they were 6 and 10 cm and at final stage they grow to 20 and 14 cm respectively. Elephant grass initial length of shoots and roots was 5 and 12, final length measured as 24 and 14 cm respectively. This was important to report, as the biomass quantity in grasses, depends upon the shoot and root length.

### Dry weight of shoots and roots after treatments

**Figure 3:**
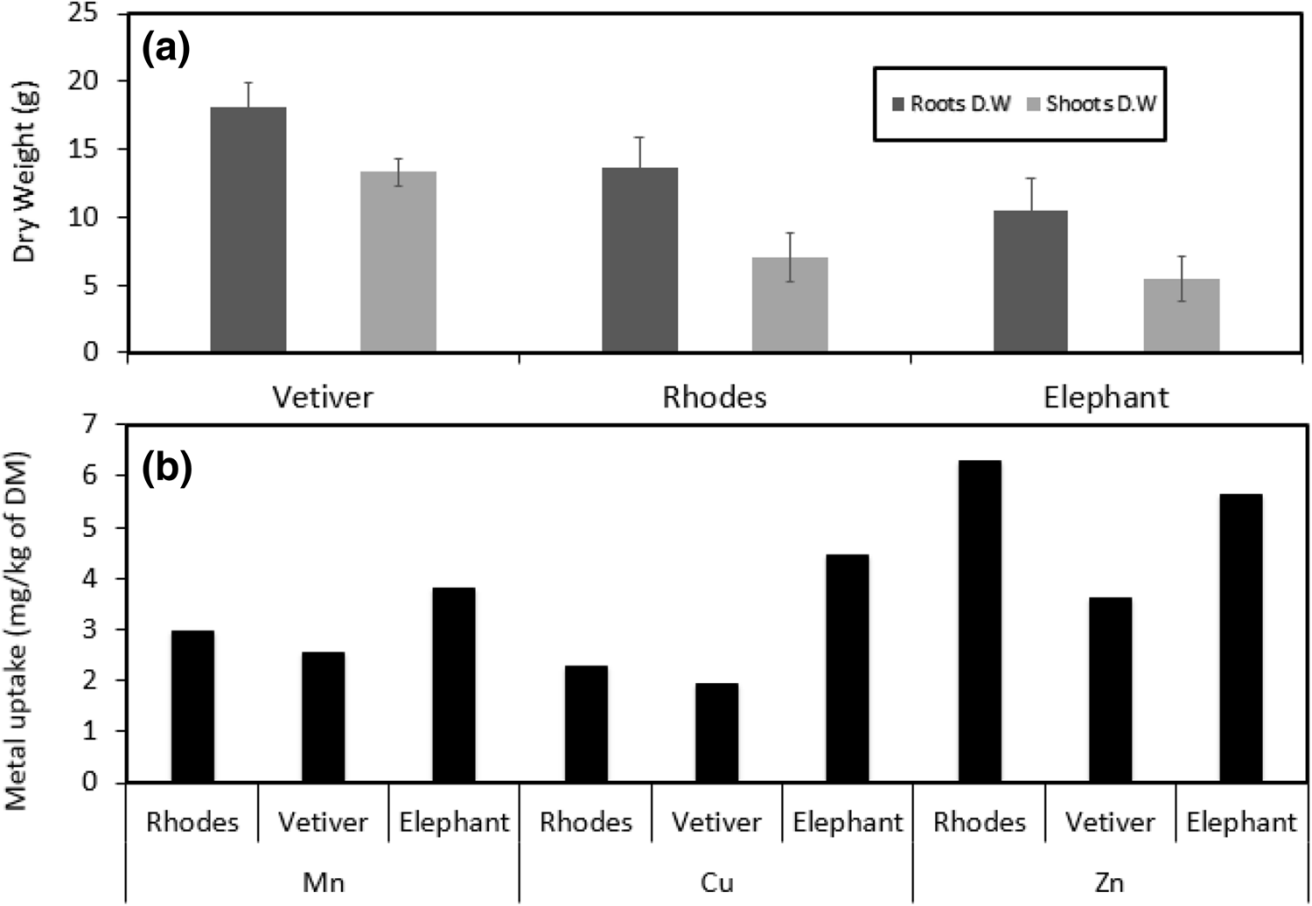
(**a**) Dry Weight of grasses shoots and roots and (**b**) uptake ratio of metals with respect to dry mass.

The weight of each dry mass (roots and shoots) is illustrated in Fig. 3.

Part (a) of Fig. 3 shows the average dry weight of Vetiver grass roots was 18 g, whereas, the weight of Rhodes and Elephant grass measured as 14 and 11 g respectively. The average dry weight of shoots was also high as 13, 7, 5 g respectively. Part (b) is representing the actual weight of metals which contribute and increase the weight of dry mass. Dry mass of Rhodes, Vetiver and Elephant grass for Mn including shoots and roots was calculated as 3 mg/kg, 3 mg/kg and 4 mg/kg respectively. In case of Cu the dry mass of Rhodes, Vetiver and Elephant grass was 2, 2, and 4 mg/kg respectively. For Zn the dry mass of Rhodes, Vetiver and Elephant grass was calculated as 6, 4, 6 mg/kg respectively.

The maximum biomass of Vetiver grass shoots and roots were at 50% leachate strength which is 37.44 g. The dry weight of root and shoot part is 21.56 and 15.65 respectively. In Elephant grass the maximum biomass produced in 30% leachate strength and bloom very well. The dry weight of biomass was noted 22.76 g whereas the mass of shoot and root was 8.35 and 14.41 g respectively. Rhodes grass was grown at 10% leachate strength and observed a dry weight of 30.63 g where roots and shoots contain 11.87 and 18.86 g respectively.

### Nutrients and heavy metal uptake by grasses

**Figure 4:**
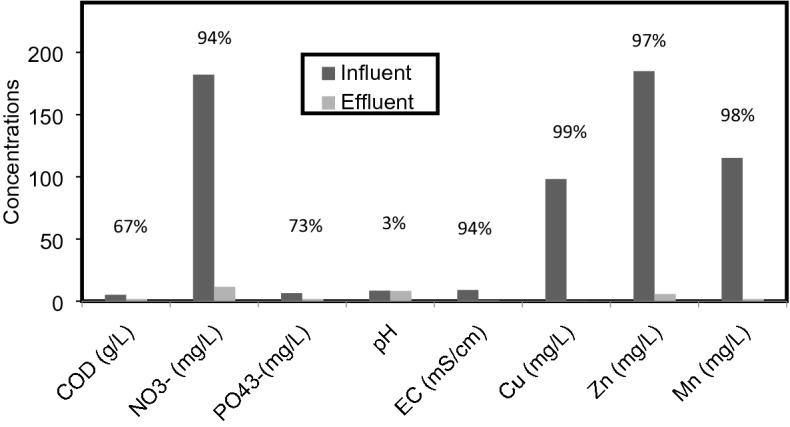
The Analysis of Leachate Contaminants before and after the Treatment of Leachate Using grasses. (The percentages shown on each column represent the amount of each contaminant accumulated by the system).

After the completion of four months plant growth in hydroponic pots using diluted leachate concentrations, analysis of various parameters studied are shown in Fig. 4. Influent and effluent concentrations of EC, pH, COD, NO3, PO4 and heavy metals along with maximum removal efficiency (shown on top) is given after removal of grasses.

The analysis for both treated and untreated leachate showed average pH values of all leachate strength, for untreated leachate it remained alkaline (pH 8.5) and slightly reduced up to 3% after treatment (pH 8.25). However, electrical conductivity reduced by almost 94% from its initial concentration. The average concentration of COD before treatment was calculated 5163.6 and reduced to 1715.2 mg/L after treatment which showed a reduction of 66.8% of COD. Similar outcome has been reported earlier^5,13^ which were 61 and 63.11% respectively. In a hydroponic experiments, investigated^24,25^ showed that vetiver grass has the ability to survive in leachate with COD of 750 mg/L to 2600 mg/L and this research showed that vetiver grass could still survive in the 100% leachate strength in which the COD value was 6930, which is a high COD value. Thus grasses are proved to be an excellent pollution resistant, bio resource and could be used to alleviate the problem of soil contamination.

**Figure 5:**
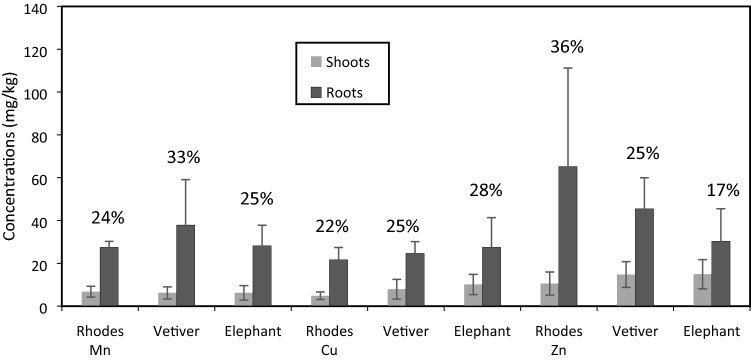
Graphical representation of different Metals uptake by Shoots and Roots of different grasses. (The percentages shown on each histogram, express the percentage of metal accumulated in shoots in terms of total accumulation).

The removal of heavy metals concentration from municipal solid waste leachate through different grasses is shown in Fig. 5.

This figure illustrates that the highest concentration of Zn was found in Rhodes grass roots. The mean concentration of Zn in roots of Rhodes grass was 36.39% (65.195 mg/kg) whereas in Vetiver and Elephant grass the average concentration was 25.37% (45.45 mg/kg) and 16.85% (30.36 mg/kg) in roots respectively. The findings have significance as Rhodes grass is not used for heavy metal removal before. Rhodes grass roots uptake high concentration of Zn than the of Vetiver and Elephant grass therefore, it should be placed near dumping cites. However, in case of Rhodes grass shoots the uptake was four times less than shoot of Vetiver and Elephant grass. The proportion of Zn in shoots and roots of Elephant grass was almost same and maximum translocation happen in shoots than Vetiver and Rhodes grass but lesser accumulation in roots than others. Thus Elephant grasses should be placed near mining sites to uptake zinc metals from the dust^26^. It was confirmed that these grass species act as metals accumulator and survived in harsh condition of high COD, EC and metal concentrations present in municipal solid waste leachate the process may be attributed transform of the metals in to inert form as reported by Kafil^27^.

In case of copper, less proportion was absorbed and translocated in roots and shoots of vetiver where Elephant grass uptake copper more efficiently than Rhodes and Vetiver grass. Similar results have been reported that the Elephant grass is more tolerant against copper^15^ and large portion accumulates in their root system. The average uptake of Elephant grass for copper was 28% (27.5 mg/kg) in roots whereas Vetiver and Rhodes grass uptake 25% (24.63 mg/kg) and (21.64 mg/kg) 22% respectively. However, in shoots of Elephant grass the trend of average uptake was the same as 10.12 mg/kg maximum then shoots of Vetiver and Rhodes grass as 7.9 mg/kg and 4.855 mg/kg respectively.

The accumulation of all heavy metals in Vetiver roots is much higher than in shoots^7,28–32^. Because it is highly tolerant to many heavy metals^33^. Mn was found maximum in roots of Vetiver grass than the shoots. The concentration of Mn in roots of Vetiver, Elephant and Rhodes grass was 33% (37.88 mg/kg), 25% (28.205 mg/kg) and 24% (27.45 mg/kg) respectively. This may be due to high cellulosic/lignin content of the grass^34^.

In this work, nitrate and phosphate were reduced by 93.7% and 73%, respectively after the treatment of leachate. The average removal of heavy metals such as zinc, copper and manganese were from 184.952 to 5.833 mg/L, 98.098 to 0.103 mg/L, and 115.209 to 1.709 mg/L respectively. The average removal of Zn, Cu and Mn from municipal solid waste (msw) leachate was 96.85%, 99.9% and 98.52% respectively are shown in Fig. 5.

### Mass Balance Of Metals

The inflow and out flow of metals was analysed before and after the treatment. The inflow of Mn, Cu, and Zn was observed as 115.21, 98.10, and 184.95 mg respectively. The percentage uptake of Mn by Rhodes, Vetiver and Elephant grass were 29.68% (34.20 mg), 38.28% (44.10 mg), respectively. Percentage Cu uptake by Rhodes, Vetiver and Elephant grass were 27.01% (26.50 mg), 33.13%(32.50 mg), and 38.33% (37.60 mg) respectively, whereas, in case of Zn uptake by Rhodes, Vetiver and Elephant grass were 40.97% (75.77 mg), 32.57% (60.23 mg), and 24.48% (45.27 mg) respectively.

## Conclusions

In this study a new species of grass namely Rhodes was tested for its performance in the municipal solid waste generated leachate to utilize its potential of phytoremediation in comparison with already tested grass species namely Vetiver and Elephant found good performer municipal wastewater of lesser concentrated than leachate. This study was conducted under hydroponic condition showed that uptake and accumulation of Mn, Cu and Zn metals by three grasses varied in the leachate. Overall collective metals uptake (Mn, Cu, Zn) was observed as 33% for Rhodes, 35% for Vetiver, and 31% for Elephant grass. The potential grass was found to be comparable with other species. It was also confirmed that all three species can withstand extremely harsh environments (leachate concentration is many fold than wastewater) and capable to uptake heavy metals that was really astonishing.

## Data Availability

On request.
